# Sugarcane growth and nutrition levels are differentially affected by the application of PGPR and cane waste

**DOI:** 10.1002/mbo3.617

**Published:** 2018-04-13

**Authors:** Roberta M. Santos, Saveetha Kandasamy, Everlon Cid Rigobelo

**Affiliations:** ^1^ Agricultural and Livestock Microbiology Post ‐ Graduation Program School of Agricultural and Veterinarian Sciences São Paulo State University (UNESP) Jaboticabal Brazil; ^2^ A&L Biologicals Agroecological Research Services Centre London ON Canada

**Keywords:** *Bacillus pumilus*, *Bacillus subtilis*, filter cake, plant growth promotion, sugar cane, vinasse

## Abstract

Mineral and organic fertilization can be optimized by using rhizobacteria which increases dry matter, yield, and nutrients in the soil and plant, among the other biological inputs. However, the discovery of single microbes or a consortium that can benefit plants has been a challenge. In this context, this study aimed to evaluate the effects of *Bacillus subtilis* and *Bacillus pumilus* combined with mineral fertilization and sugar and alcohol industry by‐products in presprouted and the initial growth phase of sugar cane seedlings. The study was carried out in two phases. Phase 1 included presprouted seedlings with T1 =  untreated control, T2 =  *B. subtilis,* T3 =  *B. pumilus*, and T4 =  *B. subtilis + B. pumilus* treatments. Phase 2 included the same treatments with four types of fertilization: F1 =  mineral fertilization, F2 =  mineral fertilization + vinasse, F3 =  mineral fertilization + filter cake, and F4 =  mineral fertilization + filter cake compost. Of the phase 1 treatments, T2 (*B. subtilis)* was the best promoter of root growth and the total dry matter compared to the control with an increase of 23.0% compared to the control. In phase 2, *B. pumilus* application, increased the total dry matter by 13%, the number of tillers by 37%, and the diameter of the tillers by 48% when combined with mineral fertilization. The combined application of *B. subtilis* and *B. pumilus* increased the phosphorus content by 13% in soil treated with mineral fertilization and filter cake compost. The results of the this study strongly suggest that the use of *B. subtilis* and *B. pumilus* together with these by‐products can improve soil fertility parameters and decrease adverse effects associated with vinasse fertilization, in addition to providing shoot and root growth and providing collective synergy for a high yield of sugarcane production with environmental benefits.

## 1 INTRODUCTION

Brazil is the world′s largest producer of sugarcane (*Saccharum* spp.), an important crop of the biofuels sector, with the potential for ethanol production and the generation of by‐products of industrial interest (De Abastecimento‐Conab, 2016). There is a high level of investment in cane production, including the generation of presprouted seedlings (PSS), the multiplication of plants in the nursery, uniform planting, and maintaining high standards of phytosanitation, etc., (Gazola, Cipola Filho, & Júnior, 2017).

In addition to using the PSS system, the use of bacterial inoculants is an alternative to achieve sustainable plant production, with less use of mineral fertilizers (Lesueur, Deaker, Herrmann, Bräu, & Jansa 2016. Studies have shown the benefits of using plant growth‐promoting rhizobacteria (PGPR) in sugarcane to increase the dry matter yield and productivity (de Oliveira, de Canuto, Urquiaga, Reis, & Baldani, 2006; Schultz et al., 2012).

Several isolates of *Bacillus*, especially *Bacillus subtilis* directly affect plant growth promotion, by the mechanisms such as acting as nitrogen fixers, as well as producers of indol butyric acid (IBA) (Araujo, Henning, & Hungria, 2005), indol acetic acid (IAA), and siderophores (Puente, Bashan, Li, & Lebsky, 2004), in addition to biocontrol activity (Ali, Charles, & Glick, 2014; Hanif et al., 2015; Oslizlo et al., 2015) and activities of protein expression relevant to the pharmaceutical and industrial sector (Guan et al., 2015). *Bacillus subtilis* has such varied functional capacities, which suggests that it has a versatile potential to be used as a plant growth promoter.

Another species with the potential to be used as plant growth promoter is *Bacillus pumilus*, which is a gibberellic acid producer, and has been shown to stimulate the growth and development of tree species such as *Alnus glutinosa* (GutiérreziMañero et al., 2001) and *Pinus pinea* (Probanza, Garcıa, Palomino, Ramos, & Mañero, 2002). It is additionally a chitinase producer, which makes it a promising antagonist to strong, economically important agricultural pathogens such as *Fusarium solani* and *Aspergillus niger* (Gurav, Tang, & Jadhav, 2017; Rishad, Rebello, Shabanamol, & Jisha, 2017).

The capability of *Bacillus* to produce durable endospores, make this genre more promising in the biofertilization business, competing with current widely used agrochemicals (Wu, Cao, Li, Cheung, & Wong, 2005).

Sugar and ethanol production generate high amounts of by‐products as waste. Filter cake, vinasse, boiler ash, and cane straw are considered the main by‐products because they have a high added value, a high potential for use in agriculture, and can be used as alternative fertilizers. Vinasse is a potential source of organic matter, with nutrients such as K, N, Ca, and Mg. It also improves water retention and can serve to increase sugarcane yield (Prado, Caione, & Campos, 2013). Filter cake is rich in organic phosphorus, which is released slowly by mineralization and the action of soil microorganisms (Almeida Júnior, do Nascimento, Sobral, da Silva, & Gomes, 2011). Compost production by combining filter cake with plaster, boiler ash, and straw adds value to the by‐product by improving the nutrient concentration and reducing the water content (da Silva, Teles, & Júnior, 2016).

Every year, the cane industry accumulates millions of tons of by‐products such as vinasse and filter cake. Moreover, because these by‐products are rich in phosphorus and potassium, they are returned to cane production as biofertilizers. Additionally, PGPR has been used in sugarcane production to reduce the necessity for mineral fertilization, thus reducing the cost of production. However, there is a dearth of studies comparing and combining the use of by‐products and bacterial inoculants on cane growth. The purpose of this study was to optimize the possibilities for the use of cane by‐products with bacterial inocula for sugarcane production.

## 2 EXPERIMENTAL PROCEDURES

This study has been conducted in two phases: Phase 1 was carried out in the greenhouse with PSS to evaluate the respective action of *B. subtilis* and *B. pumilus* on growth promotion. Phase 2 was performed outdoors by planting PSS from phase 1 in vases with a 50‐liter capacity. This served to evaluate the synergistic effect of the same bacterial inocula and cane by‐products such as vinasse, filter cake, and filter cake compost for sugarcane crop production using cane SP80‐3280 PSS.

The isolates of *B. subtilis* and *B. pumilus* came from the collection of the laboratory of Soil Microbiology of the Universidade Estadual Paulista, Câmpus de Jaboticabal. Both strains were isolated from grassy soil of the Farm of Unesp. The strains were identified by automatic sequencing of the 16 S ribosomal gene and stored as lyophilized in Brain Heart Infusion (BHI) medium in the freezer at temperature of − 20°C. The strains were reconstituted by adding 25 ml of BHI medium to 2 g of each strain. The strains were kept in a microbiological incubator for 24 hr at 28°C and 150 rotations per minute, before using in subsequent assays.

### 2.1 Phase 1

Phase 1 was carried out in a greenhouse located in Pradópolis (21º 21′ 34″ S, 48º 03′ 56″ W and 538 m altitude). The PSS were generated in plastic planting cells, and the duration of the experiment was 60 days (from November 2015 to January 2016). The design included randomized blocks with four repetitions, and the treatments were T1 =  no inoculum, T2 =  *B. subtilis*, T3 = *B. pumilus*, and T4 =  *B. subtilis* and *B. pumilus*.

Planting and the subsequent experiment were carried out by following the recommendations for minor changes outlined by Landell et al. (2012). Briefly, the buds were planted directly in the planting cells. There were 128 seedlings in total. Of these, 64 were used in phase 1, and another 64 were used in phase 2.

A microbial inoculum was prepared by individually adding *B. subtilis* and *B. pumilus* with an inoculation loop into Erlenmeyer flasks containing Brain Heart Infusion (BHI) broth. The inocula were maintained in an incubator shaker at 28°C and 150 rpm for 24 hr. Then the culture concentrations were measured and adjusted to 5.7 × 10^7^ colony‐forming units (CFU) m/L for *B. subtilis* and 1.4 × 10^8^ CFU m/L for *B. pumilus*. The concentrations were standardized according to the generation time of each bacterial species (Souza et al., 2016).

The inoculation was carried out after 15 days of PSS growth and applied to the soil via pipette by adding 2 ml of inoculum per cell for a total of 4 ml (1:1) per cell for the treatment using both bacterial species. The control plants received no inoculum.

After 60 days (at the end of phase 1), the total bacterial load present in the substrate was counted using Standard Methods Agar (SMA) (Acumedia Brazil) with the addition of 10 g of the substrate to 100 ml of sodium pyrophosphate saline solution. This was followed by plating 0.1 ml of a 10^−4^ dilution after one hour of agitation (Vieira & Nahas, 2005).

The plants were carefully harvested from the cells for the root dry matter (RDM) and shoot dry matter (SDM) measurements. The aerial and root parts were separated by cutting at the collar region and were washed to remove adhering substrate (soil medium along with by‐product treatments) in which the PSS had been cultivated. Then, the parts were placed in paper bags and dried in the green house at 65°C to constant weight. Finally, the individual RDM and SDM were weighed on a semianalytical balance, and the weight was recorded. To obtain the total dry matter (TDM), the RDM and SDM were summed.

### 2.2 Phase 2

Phase 2 was conducted for 60 days (from January to March 2016) outdoors in Jaboticabal city—Sao Paulo State (21º 14′ 05″ S, 48º 17′ 09″ W, and 615 m altitude) by transferring the PSS from phase 1 to vases. Therefore, at the end of phase 2, the plants were 120 days old.

The experimental design included randomized blocks with four repetitions as a 4 × 4 factorial using 64 pots. Both factors were composed of four treatments. The factor I treatments included bacterial inoculum: T1 =  no inoculum, T2 =  *B. subtilis*, T3 =  *B. pumilus*, and T4 =  *B. subtilis* + *B. pumilus*. The factor II treatments included cane by‐products and mineral fertilizers viz: F1 =  mineral fertilization (MF), F2 = fertilization + vinasse (FV), F3 =  MF + filter cake (MF+FC), and F4 =  MF+ filter cake compost (MF+FCC).

Bacterial inoculation for factor 1 utilized the same concentrations and methodology as described for phase 1.

Six inoculations were performed in total; the first four were weekly following the transfer of the PSS to the vases, and the last two were performed biweekly. Inoculants were applied to soil with a pipette. The inoculum (5 ml) was transferred at respective concentrations of 5.7 × 10^7^ and 1.4 × 10^8^ CFU m/L for *B. subtilis* and *B. pumilus*. The inoculum quantity was higher in phase 2 than in phase 1 because the vases were large, and the soil volume was greater, as well as because of precipitation, irrigation, and the plant growth stage during this phase.

Plant biometric data were obtained by counting all of the tillers per vase, measuring the height of the tillers using a graduated ruler from plant base up to leaf+1, according to Kuijper systems, and measuring tiller diameter near the soil using a pachymeter. Because there was more than one tiller per vase, the values of the height and diameter were summed, and the means were calculated. The RDM, SDM, and TDM were measured using methodology described previously.

For the soil analysis, samples were collected from the plant rhizosphere. The total bacterial CFU were measured using SMA medium (Buchbinder, Baris, & Goldstein, 1953) by dilution plating. The ammonium and nitrate levels were measured according to Keeney and Nelson (1982) in which the extraction was carried out with the aid of potassium chloride (KCl), followed by distillation with magnesium oxide (MgO) for the ammonium and sulfamic acid (H_3_NSO_3_), and by the method of Devarga for the determination of nitrate content. The titration sulfuric acid was used for ammonium and nitrate. Soluble phosphorus was measured by following the protocol developed by Murphy and Riley in 1986 (modified from Watanabe & Olsen, 1965).

After drying in a plant oven at 65°C (Tedesco, Gianello, Bissani, Bohnen, & Volkweiss, 1995), the nitrogen content was measured according to Keeney and Nelson (1982), and nitrogen distillation was performed as outlined by Kjeldahl, using CuSO_4_ as a catalyst.

The phosphorus content was measured according to Sarruge and Haag (1974), in which the plant samples were digested with nitric acid (65%) and perchloric acid (70%). A volume of 5.0 ml of digestion extract was pipetted, and 20.0 ml of water was added plus 4 ml of a mixture of reagents (ammonium molybdate to 5% with ammonium vanadate to 0.25%). After 5 min, a spectrometer reading was performed at 420 nm.

The data were analyzed in an analysis of variance (F test), and the means were compared by Duncan's 5% probability test using the Agro Estat 1.0 program (Barbosa & Maldonado Júnior, 2010).

## 3 RESULTS

**Figure 1:**
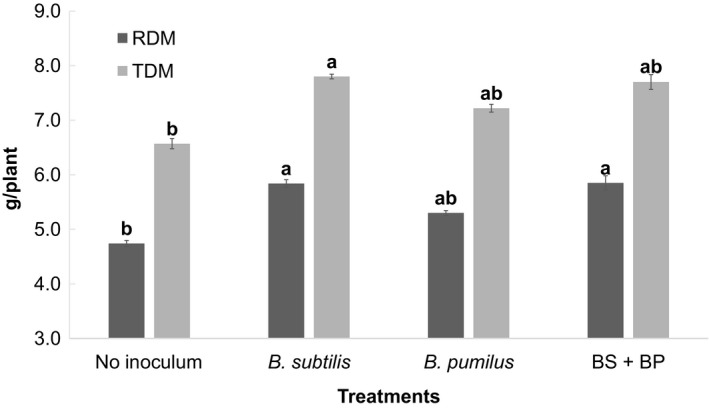
Means of root dry matter (RDM) and total dry matter (TDM) of presprouted seedlings (PSS) of sugarcane after 60 days of growth. Means followed by the same letter did not differ according to a Duncan test at 5% probability

In phase 1, inoculation of *B. subtilis* (5.84 g), *B. pumilus* (5.30 g) and a mixture of *B. subtilis* and *B. pumilus* (5.84 g) increased the plant root dry matter content compared to the control (4.74 g) (*p *>* *.05). The increase in the total dry matter content by applied microbial treatments of *B. subtilis* (6.57 g), *B. pumilus* (7.22 g), and a mixture of both microorganisms (7.7 g) were not significantly different from the control (6.57 g) (Figure 1).

**Figure 2:**
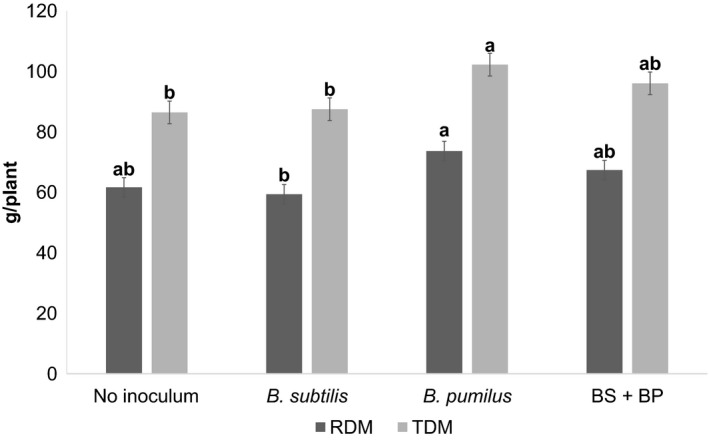
Means of root dry matter (RDM) and total dry matter (TDM) of sugarcane plants cultivated in vases 120 after planting. Means followed by the same letter did not differ according to a Duncan test at 5% probability

In phase 2, *B. pumilus* (73.7 g) promoted higher root dry matter than *B. subtilis* (59.39 g) (*p *>* *.05). The total dry matter content of *B. pumilus*‐treated plants was significantly higher (102.3 g) (*p *>* *.05), as well as *B. subtilis* (87.5 g)‐treated plants, compared to the control (86.5 g) (Figure 2). Other than this, there was no other noticeable difference in the root dry matter content of plants treated or untreated with the microorganisms.

**Figure 3:**
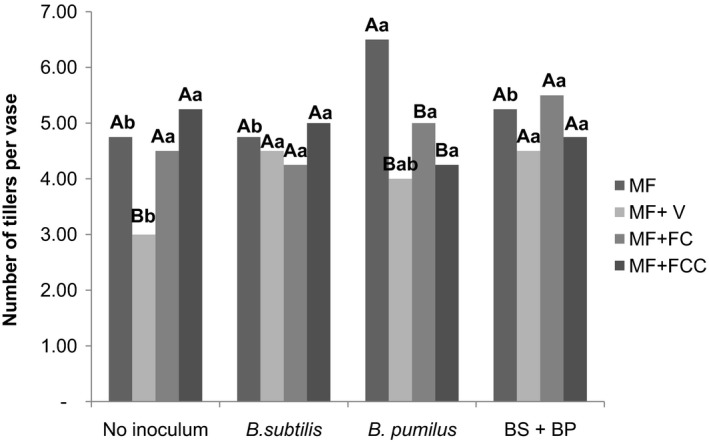
Interactions between the inoculum and the fertilization regime based on the number of tillers per vase. Uppercase letters compare means within the inocula and lowercase letters compare means within the fertilization regimes (Duncan *p* < .05). V: vinasse. FC: filter cake. FCC: filter cake compost. BS+BP= *B. subtilis* + *B. pumilus*

*Bacillus pumilus* combined with mineral fertilization produced highest number of tillers per vase (6.50) compared to the control (4.75). *Bacillus subtilis* (4.50) and a mixture of *B. subtilis* and *B. pumilus* (4.50) combined with mineral fertilization + vinasse also produced significantly more tillers than the control (3.00) (Figure 3).

**Figure 4:**
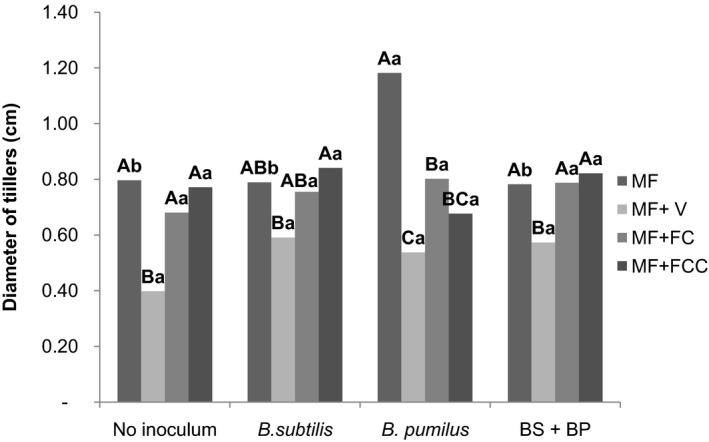
Interactions between inoculum and fertilization regime based on the diameter of tillers per vase. Uppercase letters compare means within the inocula and lowercase letters compare means within the fertilization regimes (Duncan *p* < .05). V: vinasse. FC: filter cake. FCC: filter cake compost. BS+BP= *B. subtilis* + *B. pumilus*

The diameter of the cane tillers was significantly increased with the application of *B. pumilus* combined with mineral fertilization (1.18 cm) compared to the control (0.80 cm) (*p *>* *.05) (Figure 4).

**Figure 5:**
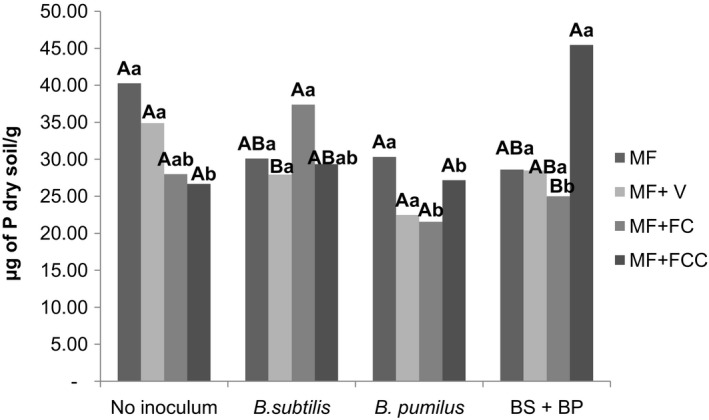
Interactions between inoculum and fertilization regime based on the soluble phosphorus concentration. Uppercase letters compare means within the inocula and lowercase letters compare means within the fertilization regimes (Duncan *p* < .05). V: vinasse. FC: filter cake. FCC: filter cake compost. BS+BP = *B. subtilis* + *B. pumilus*

The highest phosphorus content was found in soil which received a mixture of *B. subtilis* and *B. pumilus* (45.45 μg of P dry soil g^−1^) and *B. pumilus* alone (27.17 μg of P dry soil g^−1^) combined with mineral fertilization + filter cake compost applied to the plants. No significant differences were found in comparisons among the other treatments (Figure 5).

**Figure 6:**
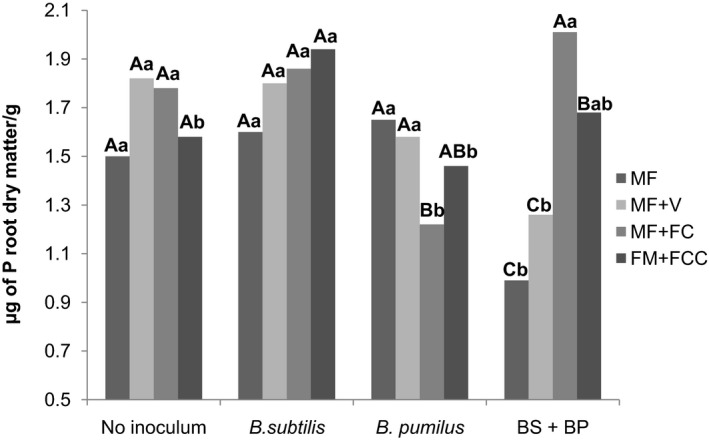
Interactions between inoculum and fertilization regime based on the phosphorus content of sugarcane roots 120 days after planting. Uppercase letters compare means within the inoculum and lowercase letters compare means within the fertilization regimes (Duncan *p* < .05). V: vinasse. FC: filter cake. FCC: filter cake compost. BS+BP= *B. subtilis* + *B. pumilus*

Inoculation of *B. pumilus* combined with mineral fertilization + filter cake decreased the P content in the roots (1.22 μg of P root dry matter g^−1^) compared to the control (1.78 μg of P root dry matter g^−1^). Interestingly, the effect was stronger in plants which received a mixture of *B. subtilis* and *B. pumilus* combined with both mineral fertilization (0.99 μg of P root dry matter g^−1^) and mineral fertilization + vinasse (1.26 μg of P root dry matter g^−1^) compared to the respective controls (1.50 μg of P root dry matter g^−1^ and 1.82 μg of P root dry matter g^−1^) (Figure 6).

**Figure 7:**
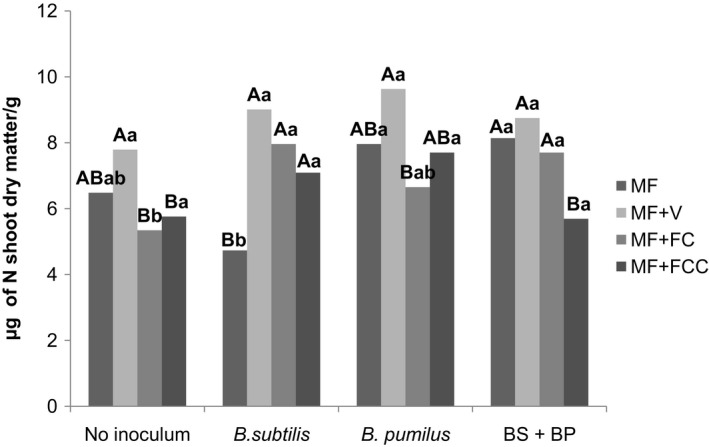
Interactions between inoculum and fertilization regime based on the nitrogen content of sugarcane roots 120 days after planting. Uppercase letters compare means within the inoculum and lowercase letters compare means within the fertilization regimes (Duncan *p* < .05). V: vinasse. FC: filter cake. FCC: filter cake compost. BS+BP= *B. subtilis + B. pumilus*

Figure 7 depicts the nitrogen content of the plant shoots. Inoculation of a mixture of *B. subtilis* and *B. pumilus* increased the nitrogen content of the plants (7.7 μg of N shoot dry matter) compared to the control (5.3 μg of N shoot dry matter) in the mineral fertilization + filter cake treatments. Additionally, a higher nitrogen content was recorded in the mineral fertilized plants inoculated with *B. pumilus* (8.0 μg of N shoot dry matter) and a mixture of *B. subtilis* and *B. pumilus* (8.1 μg of N shoot dry matter) than in the control (6.5 μg of N shoot dry matter).

## 4 DISCUSSION

In phase 1, the inoculation of *B. subtilis*,* B. pumilus* and a mixture of both microorganisms promoted an increase of root dry matter, the total dry matter content of the plants compared to the control. In phase 2, *B. pumilus* promoted the highest root and total plant dry matter content compared to *B. subtilis* and *B. pumilus* combinations and the control.

The highest number of tillers was produced by *B. pumilus* combined with mineral fertilization. The combined application of *B. pumilus* + *B. subtilis* also significantly increased the number of tillers when mineral fertilization + vinasse was added.

The diameter of the tillers was increased when the plants received *B. pumilus* compared to the control with mineral fertilizer added.

The application of *B. pumilus* provided major benefits to the plants and the soil. Although reports on the enhancement of plant growth via PGPR are widely available, information about the use of *B. pumilus* and *B. subtilis* in the presence of different fertilization regimes that use cane waste for sugarcane production is scarce. Many studies regarding *B. pumilus* report that enzymes such as endoglucanase (Ariffin et al., 2008); xylanases (Bajaj, Khajuria, & Singh, 2012; Kapilan & Arasaratnam, 2011; Kapoor, Nair, & Kuhad, 2008); and chitinase (Gurav et al., 2017) are produced. A few studies report their capacity for plant growth promotion. According to our knowledge, this study is the first report about the use of *B. pumilus* as plant growth promoter in sugarcane production along under different fertilization regimes that mainly use cane waste for sugarcane production.

Phytohormones produced by *B. pumilus* and *B. subtilis* greatly contribute to the enhancement of plant development. Phytohormones are organic substances that can promote, inhibit, or modify the growth and development of plants at low concentrations (Damam, Kaloori, Gaddam, & Kausar, 2016). Phytohormones promote root cell proliferation by overproducing lateral roots and root hairs with a concomitant increase in nutrient and water uptake (Sureshbabu, Amaresan, & Kumar, 2016). The results of the present study show that increased root and shoot dry matter and tiller number and diameter could be due to the phytohormones produced by these bacteria.

Mineral fertilization enhances plant development because the plants are nourished and rescued from a nutritional deficiency. However, approximately 40%–70% N, 80%–90% P, 50%–70% K of the total applied conventional fertilizer are lost to the environment due to variations in the soil dynamics (Fageria, 2014), and the use of *B. pumilus* and *B. subtilis* improved the efficiency of nutrient uptake by plants fertilized with mineral fertilizers, thereby promoting enhanced plant growth.

(Breedt, Labuschagne, & Coutinho, 2017) verified the use of *B. pumilus* to promote maize growth, and they recorded significant enhancement of plant growth and development and reported that the commercialization of these bacteria is a viable option. Moon, Asif, and Basharat (2017) reported that the use of *B. pumilus* increased root and shoot dry matter, as also reported in this study. Auxin produced by different *Bacillus* strains is associated with enhanced vegetative growth parameters of grasses.

A significant portion of sugarcane industry uses PSS for sugarcane cultivation. This procedure significantly reduces the seedling cane volume spend per hectare, increases the multiplication rate, improves seedling sanitation and planting uniformity, and the use of a smaller volume of material in the field with an increase in field operability (Landell et al., 2012). The positive effects of *B. pumilus* and *B. subtilis* in growth promotion and plant development in phase 1 indicate that both species could be used to reduce the time taken to produce PSS. Thus, the sugarcane industry can increase its profitability by increasing the number of seedlings formed while maintaining the same structure used to produce these PSS seedlings.

The negative effect of application of mineral fertilization + vinasse, which significantly reduced the number of tillers produced per plant, has been reversed by the application of a mixture of *B. subtilis* + *B. pumilus*. This result suggests a dual role for microbial inoculation; the simultaneous promotion of plant growth while decreasing the adverse effects of vinasse application. Certainly, the microbial mixture had a strong impact on the ability of the plants to cope with the stress caused by the vinasse. In the this study, the amount of vinasse applied to the soil followed the recommendation by Prado et al. (2013). The vinasse used in this study was analyzed and was found to contain a microbiota with a high number of microorganisms, a high K content, and no alcohol content (data not shown). Interestingly, it reduced the number of tillers produced per plant. So, further detailed studies are needed to understand the composition of the vinasse, and the causes of its adverse effect on the sugarcane seedlings.

The highest phosphorus content was found in soil which received a mixture of *B. subtilis* and *B. pumilus* combined with mineral fertilization + filter cake compost.

Phosphorus is the second most essential nutrient required by plants in adequate quantities for optimum growth. It plays an important role in almost all major metabolic processes, including energy transfer, signal transduction, respiration, macromolecular biosynthesis, and photosynthesis (Anand, Kumari, & Mallick, 2016). However, 95%–99% of phosphorus present in the soil is in insoluble, immobilized, or precipitated. Therefore, it is difficult for plants to absorb it directly. Solubilization and mineralization of phosphorus by phosphate solubilizing bacteria such as *B. pumilus* and *B. subtilis* has been reported by, Kaushal, Kumar, and Kaushal, (2017), and this study shows that it is an important state that can be achieved. Filter cake compost has been shown to be an effective type of fertilization that promotes phosphate (PO_4_) solubilization and increases P availability in the soil compared to other types of fertilization. Filter cake has been singled out for its great potential for agricultural use and has been utilized with good results as a substitute for PO_4_ mineral fertilizers in field crop production (Prado et al., 2013). Our results suggest that filter cake compost can be best employed when applied in conjunction with *B. pumilus* and *B. subtilis* microbial mixtures.

A decreased phosphorus content was recorded in the current study in sugarcane roots when both *B. subtilis* and a mixture of *B. subtilis* + *B. pumilus* were applied. This result may have occurred due to a negative feedback mechanism by high concentrations of indole acetic acid (IAA), because *B. subtilis* and *B. pumilus* are strong IAA producers, which is known to increase root area and biomass, facilitating microbial carbon uptake. Consequently, it increases the capacity of the roots to take up water and minerals as well as root development. Additionally, some studies have shown that many genes involved in phosphorus transport are downregulated by the presence of IAA in the roots.

Talboys, Owen, Healey, Withers, and Jones (2014) reported a low phosphorus content in the soil when *B. amyloliquefaciens* was used as an inoculant with mineral fertilization + filter cake compost, which supports the results of the current study, but this negative effect did not occur when other types of fertilization were used. It may be that the reduced phosphorus content in the roots was essential at the initial stage for positive bacterial behavior and their establishment in the roots. Further studies are needed to verify the effect and to strongly support the mechanism of action.

Biological nitrogen fixation is a process that accounts for approximately two‐thirds of the nitrogen fixed globally. This biological process is carried out either by symbiotic or nonsymbiotic bacteria, and it is mediated by nitrogenase Wu et al. 2005. Nitrogenase activity in the soil depends on ecological conditions in association with the specific nitrogen fixing capabilities of certain microorganisms and plant genotypes and various climatic conditions such as moisture, oxygen concentration, and the supply of organic C substrates (Shridhar, 2012). In the present study, the mineral fertilization + filter cake compost probably provided the best ecological conditions mainly because of the supply of organic C substrates to *B. pumilus* and *B. subtilis,* which allowed the expression of their potential to fix nitrogen in the roots, compared to other fertilization regimes.

Plant growth‐promoting bacteria are free‐living soil, rhizosphere, rhizoplane or phylosphere bacteria, and they are beneficial to plants under certain conditions. The participation of these bacteria in beneficial activities is associated with their enzymatic activity and their establishment in specific niches. Such favorable conditions are strongly influenced by the type of fertilization supplied. Some by‐products of the sugarcane industry are produced in high amounts, and attempts have been constantly made to use them for cane production, as a way to improve soil fertility, decrease the need for chemical fertilization, and avoid environmental pollution.

## 5 CONCLUSION

This study strongly suggests that the use of *B. subtilis* and *B. pumilus* improves the quality of cane by‐products, enhances soil fertility, and decreases the adverse effects of vinasse fertilization in addition to plant growth promotion, which is a strong evidence for the combination of microbes and cane by‐products to produce high yield and productivity in sugarcane production.

## CONFLICT OF INTEREST

The authors declare to have no conflict of interest.
